# The complete mitochondrial genome of *Mnais tenuis* Oguma, 1913 (Odonata: Calopterygidae) and its phylogenetic implications

**DOI:** 10.1080/23802359.2021.1926360

**Published:** 2021-05-13

**Authors:** Liang-Jong Wang, Meng-Hao Hsu, Chiu-Hsien Wang, Chih-Hsin Chung, Chia-Hsuan Sung

**Affiliations:** a Division of Forest Protection, Taiwan Forestry Research Institute, Taipei, Taiwan; b Division of Watershed Management, Taiwan Forestry Research Institute, Taipei, Taiwan; c Department of Forestry and Natural Resource, National Ilan University, Yilan, Taiwan; d Planning and Information Division, Fisheries Research Institute, Keelung, Taiwan

**Keywords:** Mitochondrial genome, *Mnais tenuis*, Calopterygidae, Odonata, next-generation sequencing

## Abstract

We sequenced and assembled the complete mitochondrial genome of *Mnais tenuis* from Darshi, Taoyuan County, Taiwan. The complete mitogenome of *M. tenuis* is 15,131 bp long, and contains 13 protein-coding, 22 tRNA, and two rDNA genes. Nucleotide compositions of the mitogenome of the *M. tenuis* are A: 40.08%, T: 25.47%, C: 20.38%, and G: 14.07%. The AT and GC skewness of the mitogenome sequence was 0.2228 and −0.183, showing the A-skew and C-skew. The clade including *M. tenuis* and all the other Odonata species received absolute support (100%). The phylogenetic position of Anisozygoptera is sister to Anisoptera. *Mnais* is phylogenetically close to *Psolodesmus*. Mitogenomic data from this study will provide useful information for further studies for the population genetics, speciation and conservation of *M. tenuis* in the future.

The taxonomy of the odonate genus *Mnais* Selys has been considered a difficult task (Hämäläinen 2004). There are nine species in the genus (Paulson and Schorr 2020). The genus is widespread in Eastern Asia. A singular feature in all *Mnais* species is that males have two color forms, hyaline-winged and orange-winged. Due to much local variation in each species, it is difficult to classify the various intra-generic taxa using morphological characters. DNA data provide essential evidence of species delineation within the genus (Karjalainen and Hämäläinen 2013). *Mnais tenuis* inhabits forest streams in Taiwan. Females typically lay their eggs in moss- or algae-covered stones along the stream. The flight period is from March to June (Wang 2000). *Mnais tenuis* is distributed in Taiwan and eastern China. This is the first report of complete mitochondrial sequences for the species *M. tenuis*.

The single specimen of *M. tenuis* in this study was collected in Darshi, Taoyuan County, Taiwan (N24°54′14.0″ E121°19′07.0″) in April 2017. Total genomic DNA was extracted from the legs of the adult using a QuickExtract™ DNA Extraction Solution kit (Epicentre, Madison, WI) following the supplier’s instructions. The voucher specimen (accession number: Mte2017W001) and its genomic DNA (accession number: Mte2017WGDNA001) were deposited in the Lab. of Forest Insects and Systematic Entomology, Taiwan Forestry Research Institute, Taipei, Taiwan (L. J. Wang, ljwang23@ms17.hinet.net). The voucher specimen and other specimens collected in the same stream were identified to species level by L. J. Wang based on the reference (Wang 2000). The complete mitogenome of *M. tenuis* was sequenced using the next-generation sequencing method (Illumina MiSeq, San Diego, CA). A total of 1.5 Gb next-generation sequencing paired-end reads were used to assemble the complete mitogenome sequence (Hahn et al. 2013). The CLC Genomics Workbench (QIAGEN, Hilden, Germany) was used for sequence quality analysis, data trimming, and de novo assembling. The locations of the protein-coding genes, ribosomal RNAs (rRNAs), and transfer RNAs (tRNAs) were predicted by using MITOS Web Server (Bernt et al. 2013) and identified by alignment with other mitogenomes of damselflies in the family Calopterygidae. The AT and GC skew was calculated according to the following formulas: AT skew=(A – T)/(A + T) and GC skew=(G – C)/(C + G) (Perna and Kocher 1995). The phylogenetic analyses based on Bayesian inference (BI) were performed using Mrbayes v. 3.2.4 (Huelsenbeck and Ronquist 2001) under model GTR + I+G.

The complete mitogenome of *M. tenuis* is 15,131 bp in length (GenBank accession no. MW015098), including 13 protein-coding genes, two rRNA genes, 22 tRNA genes, and one control region. The total nucleotide compositions of the *M. tenuis* mitogenome are 40.08% for A, 25.47% for T, 20.38% for C, and 14.07% for G. The AT and GC skewness of mitogenome sequence, showing the A-skew and C-skew, was 0.2228 and −0.183, respectively. The gene rearrangement of the mitogenome in *M. tenuis* is identical to the ancestral inferred insect type (Cameron 2014). We reconstructed the phylogenetic relationships including 30 Odonata species and two Plecopteran species (*Pteronarcys princeps* and *Dinocras cephalotes*) as outgroup based on 13 mitochondrial protein-coding genes (Figure 1). Nodal supports were indicated by posterior probabilities. The clade including *M. tenuis* and all the other Odonata species received absolute support (100%). The phylogenetic position of Anisozygoptera (*Epiophlebia superstes* (NC023232)) is sister to the clade including all Anisoptera species. Anisoptera, Anisozygoptera, and Zygoptera are definitely a monophyletic group based on our result. The genus *Mnais* including *M. tenuis* and *M. costalis* (AP017642 and KU817065) is a monophyletic group. *Mnais* is phylogenetically close to *Psolodesmus* in this study, consistent with the result of the previous studies (Dumont et al. 2005; Wang et al. 2019). More complete mitogenomic data from other Odonata species are needed for further studies on the phylogeny of Odonata. Mitogenomic data from this study will provide useful information for further studies for the population genetics, speciation, and conservation of *M. tenuis* in the future.

**Figure 1.**
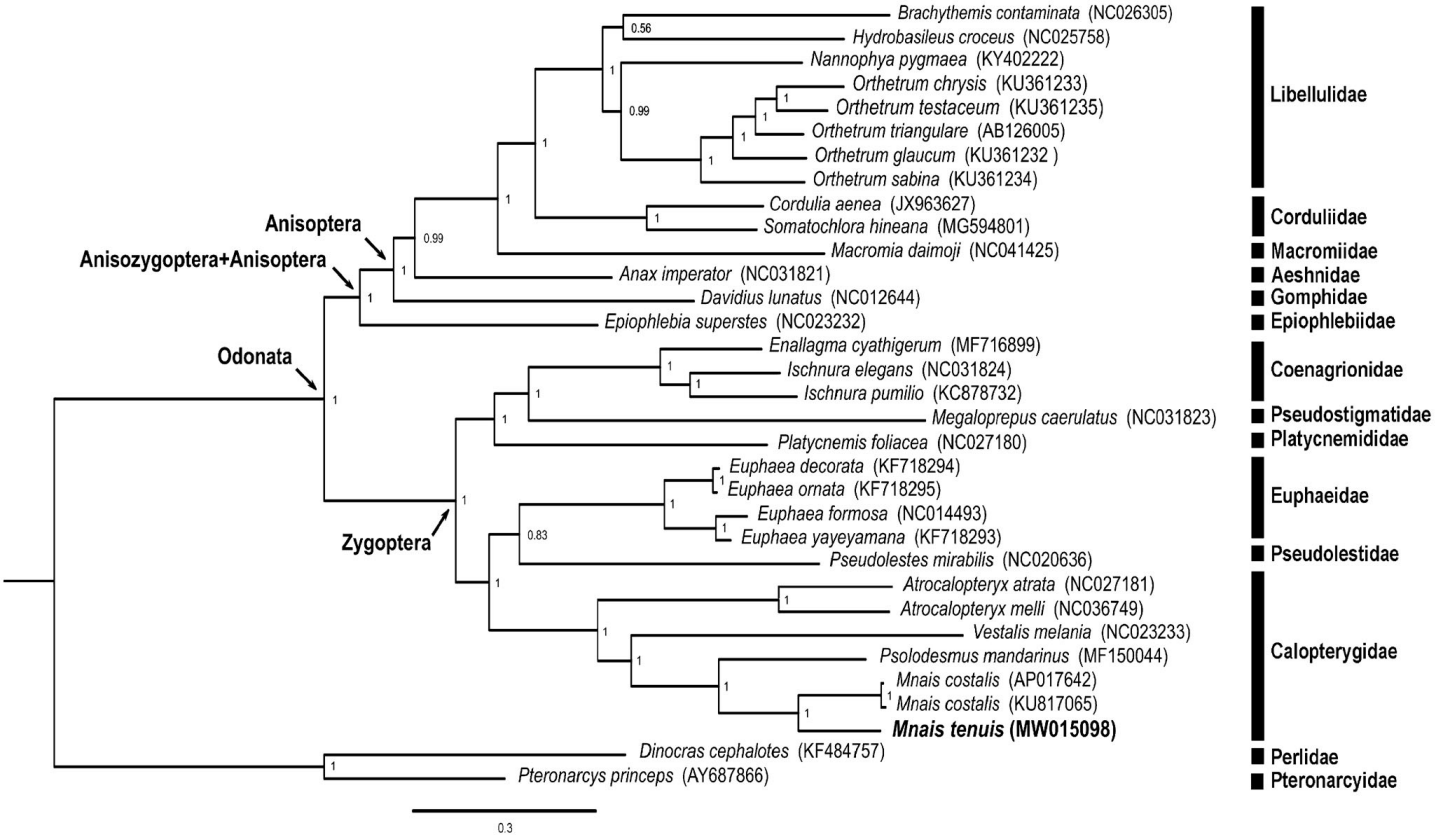
Phylogenetic tree of the 30 Odonata species including *Mnais tenuis* (in this study, MW015098) and two Plecoptera species based on the sequence of 13 protein-coding genes. The phylogenetic tree was inferred with Mrbayes v. 3.2.4 (Huelsenbeck and Ronquist 2001) under model GTR + I+G. Value on nodes indicated posterior probabilities.

## Data Availability

The genome sequence data that support the findings of this study are openly available in GenBank of NCBI (National Center for Biotechnology Information) at https://www.ncbi.nlm.nih.gov under the accession no. MW015098. The associated BioProject, SRA, and Bio-Sample numbers are PRJNA712956, SRR13921209, and SAMN18042822, respectively.
